# Comparable Norovirus Shedding in Symptomatic and Asymptomatic Individuals: A Hospital-Based Study in Bangladesh

**DOI:** 10.1093/ofid/ofag658

**Published:** 2026-10-04

**Authors:** Sharmeen Akter Urmi, Adiba Hassan, Farzana Fariha, Shahreen Rahman, Probir Kumar Ghosh, Md Mahfuzur Rahman, Faruq Abdulla, Zarin Abdullah, Rashi Gautam, Mohammad Enayet Hossain, Sayera Banu, Jan Vinjé, Sara Mirza, Mustafizur Rahman, Umesh D Parashar, Syed M Satter, Paul A Gastañaduy

**Affiliations:** Infectious Diseases Division (IDD), International Centre for Diarrhoeal Disease Research, Bangladesh (icddr,b), Dhaka, Bangladesh; Viral Gastroenteritis Branch, Division of Viral Diseases, National Center for Immunization and Respiratory Diseases, Centers for Disease Control and Prevention, Atlanta, Georgia, USA; Infectious Diseases Division (IDD), International Centre for Diarrhoeal Disease Research, Bangladesh (icddr,b), Dhaka, Bangladesh; Infectious Diseases Division (IDD), International Centre for Diarrhoeal Disease Research, Bangladesh (icddr,b), Dhaka, Bangladesh; Infectious Diseases Division (IDD), International Centre for Diarrhoeal Disease Research, Bangladesh (icddr,b), Dhaka, Bangladesh; Infectious Diseases Division (IDD), International Centre for Diarrhoeal Disease Research, Bangladesh (icddr,b), Dhaka, Bangladesh; Infectious Diseases Division (IDD), International Centre for Diarrhoeal Disease Research, Bangladesh (icddr,b), Dhaka, Bangladesh; Department of Epidemiology, Biostatistics and Occupational Health, McGill University, Montreal, Quebec, Canada; Viral Gastroenteritis Branch, Division of Viral Diseases, National Center for Immunization and Respiratory Diseases, Centers for Disease Control and Prevention, Atlanta, Georgia, USA; Infectious Diseases Division (IDD), International Centre for Diarrhoeal Disease Research, Bangladesh (icddr,b), Dhaka, Bangladesh; Infectious Diseases Division (IDD), International Centre for Diarrhoeal Disease Research, Bangladesh (icddr,b), Dhaka, Bangladesh; Viral Gastroenteritis Branch, Division of Viral Diseases, National Center for Immunization and Respiratory Diseases, Centers for Disease Control and Prevention, Atlanta, Georgia, USA; Viral Gastroenteritis Branch, Division of Viral Diseases, National Center for Immunization and Respiratory Diseases, Centers for Disease Control and Prevention, Atlanta, Georgia, USA; Infectious Diseases Division (IDD), International Centre for Diarrhoeal Disease Research, Bangladesh (icddr,b), Dhaka, Bangladesh; Viral Gastroenteritis Branch, Division of Viral Diseases, National Center for Immunization and Respiratory Diseases, Centers for Disease Control and Prevention, Atlanta, Georgia, USA; Infectious Diseases Division (IDD), International Centre for Diarrhoeal Disease Research, Bangladesh (icddr,b), Dhaka, Bangladesh; Viral Gastroenteritis Branch, Division of Viral Diseases, National Center for Immunization and Respiratory Diseases, Centers for Disease Control and Prevention, Atlanta, Georgia, USA

**Keywords:** asymptomatic viral infections, Bangladesh, gastroenteritis, norovirus, prolonged shedding

## Abstract

**Background:**

The frequent detection of norovirus in the stool of healthy people may be due to prolonged norovirus shedding after recovery from illness or asymptomatic infections. We describe norovirus shedding patterns in symptomatic and asymptomatic persons in Bangladesh.

**Method:**

We enrolled acute gastroenteritis (AGE) cases and non-AGE controls from 2 hospitals from January to September, 2023. Fecal samples were tested for norovirus by rapid antigen testing and confirmed by real-time reverse transcription-quantitative polymerase chain reaction (RT-qPCR). Positive persons were retested at days 20, 40, 60, and 120 by RT-qPCR. Shedding duration was calculated as the number of days from enrollment to the midpoint between the last norovirus-positive and first norovirus-negative specimens. Cycle threshold (Ct) values served as an inverse proxy for the viral load.

**Results:**

Among 16 persons confirmed infected with norovirus (11/32 AGE cases; 5/28 non-AGE controls), a total of 74 stool samples were collected from enrollment through follow-up, of which 28 (38%) were norovirus positive. Of these, 20 (71%) were typed as GII.4 Sydney[P16], 1 as GII.3[P16], and 7 as GII untypeable. The median duration of norovirus shedding was similar between AGE cases (10 days, interquartile range [IQR], 9.5–29 days) and asymptomatic controls (9.5 days, IQR, 9.5–49 days). Norovirus Ct values varied substantially between persons and overlapped significantly among AGE cases and non-AGE controls.

**Conclusions:**

Norovirus shedding quantity and duration were similar between AGE cases and asymptomatic controls, complicating the attribution of norovirus infection to AGE in Bangladesh. Larger longitudinal studies would more accurately characterize norovirus shedding in resource-limited settings.

## Teaser

We studied norovirus shedding among symptomatic and asymptomatic persons in Bangladesh and found similar duration and viral loads, complicating attribution of diarrhea to norovirus and interpretation of Ct values in endemic settings.

Noroviruses are a leading cause of acute gastroenteritis (AGE) globally in all age groups [1] and account for ∼50% of all foodborne outbreaks and >90% of all viral foodborne outbreaks worldwide [2, 3]. Noroviruses are nonenveloped, single-stranded RNA viruses with an approximately 7.5 kb genome comprising 3 open reading frames (ORFs). ORF1 encodes nonstructural proteins, including the RNA-dependent RNA polymerase (RdRp), while ORF2 encodes the major capsid protein (VP1), and ORF3 encodes the minor (VP2) capsid proteins [4, 5]. Noroviruses are genetically diverse and are classified into genogroups (G), genotypes, and polymerase-types (P-types) based on phylogenetic analyses and VP1 and RdRp sequences [5, 6]. Human infections are predominantly caused by GI and GII noroviruses [7], and GII.4 has historically been the dominant genotype globally, with multiple new variants emerging over time [8].

The frequent detection of norovirus in stool specimens of asymptomatic persons complicates efforts to accurately estimate the virus' contribution to acute diarrhea in highly endemic settings. For example, in a previous hospital-based case–control study in Bangladesh, norovirus prevalence was higher among non-AGE controls (15%) than among AGE cases (9%) [9]. Such a finding is often ascribed, in part, to prolonged shedding of norovirus after recovery from illness or to asymptomatic infections [10].

Persons ill with norovirus can shed high amounts of the virus in feces, reaching peak levels within 2–5 days after infection [11]. Viral particles are often shed for 2–4 weeks, even after symptoms have resolved, and occasionally, it may take up to 2–4 months for the virus to be undetectable in stool specimens [11–18]. Less is known about the duration of shedding among persons infected with norovirus who never develop symptoms [13, 14, 17], and few studies have compared the concentration of fecal viral particles in symptomatic and asymptomatic persons over the course of their infections [19]. Infected persons who recover and transition to asymptomatic status, and those who remain symptom-free during infection, can serve as a viral reservoir and contribute to ongoing transmission; thus, data on the duration of shedding in areas with a high burden of norovirus is important. Better understanding the dynamics of norovirus shedding in symptomatic and asymptomatic persons can help optimize the selection of cases and controls in studies aimed at establishing the clinical burden of norovirus, as well as evaluate the utility of viral load in assessing norovirus pathogenicity.

To understand how prolonged shedding influences the detection of norovirus in asymptomatic persons and assess the use of viral load in discerning between symptomatic and asymptomatic norovirus infections, we investigated the duration and concentration of norovirus shedding in people who are symptomatic and asymptomatic in Bangladesh.

## METHODS

### Selection of Acute Gastroenteritis Cases and Nonacute Gastroenteritis Controls and Collection of Clinical Data

This study was conducted in 2 urban tertiary-care hospitals in Bangladesh from January 18 to 30 September 2023. A government hospital in Rajshahi and a private hospital in Kishoreganj were selected to ensure inclusion of people from different socioeconomic backgrounds. Given the high volume of people seeking care for diarrheal illness at Bangladeshi hospitals, we employed a systematic sampling approach by which every 10th person admitted with AGE was selected for enrollment. Acute gastroenteritis was defined as the occurrence of 3 or more liquid or semiliquid stools, or 1 or more episodes of forceful vomiting, within a 24-hour period, with symptoms lasting less than or equal to 7 days. For each AGE case, a person with non-AGE admitted to the same hospital within 30 days, and of the same sex and age group (0–4, 5–17, and ≥18 years), was selected for enrollment. Non-AGE was defined as having no symptoms of AGE during the 14 days preceding enrollment. Written informed consent was obtained from AGE cases and non-AGE controls upon enrollment. Clinical data on AGE symptoms were collected at enrollment and during each sample collection time-point.

### Specimen Collection and Norovirus Testing

Approximately 4 mL (4 g) of bulk stool was collected from AGE cases and non-AGE controls at enrollment and each sampling day. Enrollment stool specimens were tested for norovirus using a rapid antigen detection test (RDT; Standard F Norovirus Antigen Plus rapid, qualitative fluorescent immunoassay, SD Biosensor Inc.) [20]. Every 2 weeks, RDT-positive specimens were sent to the virology laboratory of the International Center for Diarrhoeal Disease Research (icddr,b) for confirmatory real-time reverse transcription-quantitative polymerase chain reaction (RT-qPCR) testing [21]. Acute gastroenteritis cases and non-AGE controls who tested RT-qPCR positive were followed at home by trained field assistants on days 20, 40, 60, and 120 postenrollment for further stool collection and RT-qPCR testing. All stool specimens were preserved at −70°C prior to RT-qPCR testing at the virology laboratory of icddr,b. Reverse transcription-quantitative polymerase chain reaction was performed using oligonucleotide primers and probes targeting the ORF1–ORF2 junction region, as published previously [21]. Reactions were performed using a CFX96 Touch Real-Time PCR Detection System (Bio-Rad Laboratories). Cycle threshold (Ct) values were used as a semiquantitative indicator of relative norovirus RNA abundance, with lower Ct values indicating relatively higher amounts of target RNA.

### Sequence Analyses and Genotyping

For genotyping, a sequence including the 3′-ORF1 and 5′-ORF2 regions was amplified [22] and sequenced using Oxford Nanopore Technology (MinION platform), generating amplicons of 557 base pairs. Raw sequence reads were aligned to a reference norovirus sequence (GenBank accession no. NC_039547.1) to guide sequence assembly. The assembled sequences were further analyzed using BioEdit v7.2 [23] to ensure sequence accuracy and quality. Finally, the consensus sequences were genotyped using the US Centers for Disease Control and Prevention (CDC) Human Calicivirus Typing Tool (https://calicivirustypingtool.cdc.gov/).

### Duration of Norovirus Shedding

Norovirus shedding duration was defined as the interval (in days) from the first norovirus-positive specimen to the midpoint between the last norovirus-positive specimen and first norovirus-negative specimen. These additional days (following the last norovirus-positive specimen) were included because sampling was done at prespecified intervals, leaving the exact end of shedding unknown. A positive specimen following a negative specimen, or a different genogroup or genotype, was considered a new infection. Shedding duration was calculated only for initial infections.

Sensitivity analyses on the duration of shedding were performed using 2 alternative definitions: (1) a narrow interval defined as the interval (in days) from the first norovirus-positive specimen to the last norovirus-positive specimen and (2) a wide interval defined as the interval (in days) from the first norovirus-positive specimen to the first norovirus-negative specimen.

### Statistical Analysis

Demographic and clinical characteristics of confirmed norovirus-positive AGE cases and non-AGE controls were summarized using proportions for categorical variables and medians and interquartile ranges for continuous variables. The duration of norovirus shedding was estimated by Kaplan–Meier survival curves, and differences in the median duration of shedding between AGE cases and non-AGE controls were compared using a log-rank test. We compared the viral load between AGE cases and non-AGE controls by plotting the distribution of Ct values using kernel density plots; these comparisons were made with Ct values obtained at the following sampling timeframes: at enrollment, during 20–60 days of follow-up, and from enrollment to 120 days of follow-up.

## RESULTS

We tested 676 AGE cases and 562 non-AGE controls, with 32 AGE cases and 28 non-AGE controls testing positive for norovirus by RDT. Of these, RT-qPCR confirmed norovirus in 12 AGE cases and 5 non-AGE controls. After 1 norovirus-positive AGE case declined to provide follow-up specimens, the final analysis included 11 norovirus-positive AGE cases and 5 norovirus-positive non-AGE controls.

The demographic and clinical characteristics of the 11 AGE cases and 5 non-AGE controls are presented in Table 1. The median age was similar between AGE cases (11 months, interquartile range [IQR], 6–96 months) and non-AGE controls (12 months, IQR, 6–23 months), as was the overall age distribution (Figure 1).

**Figure 1.**
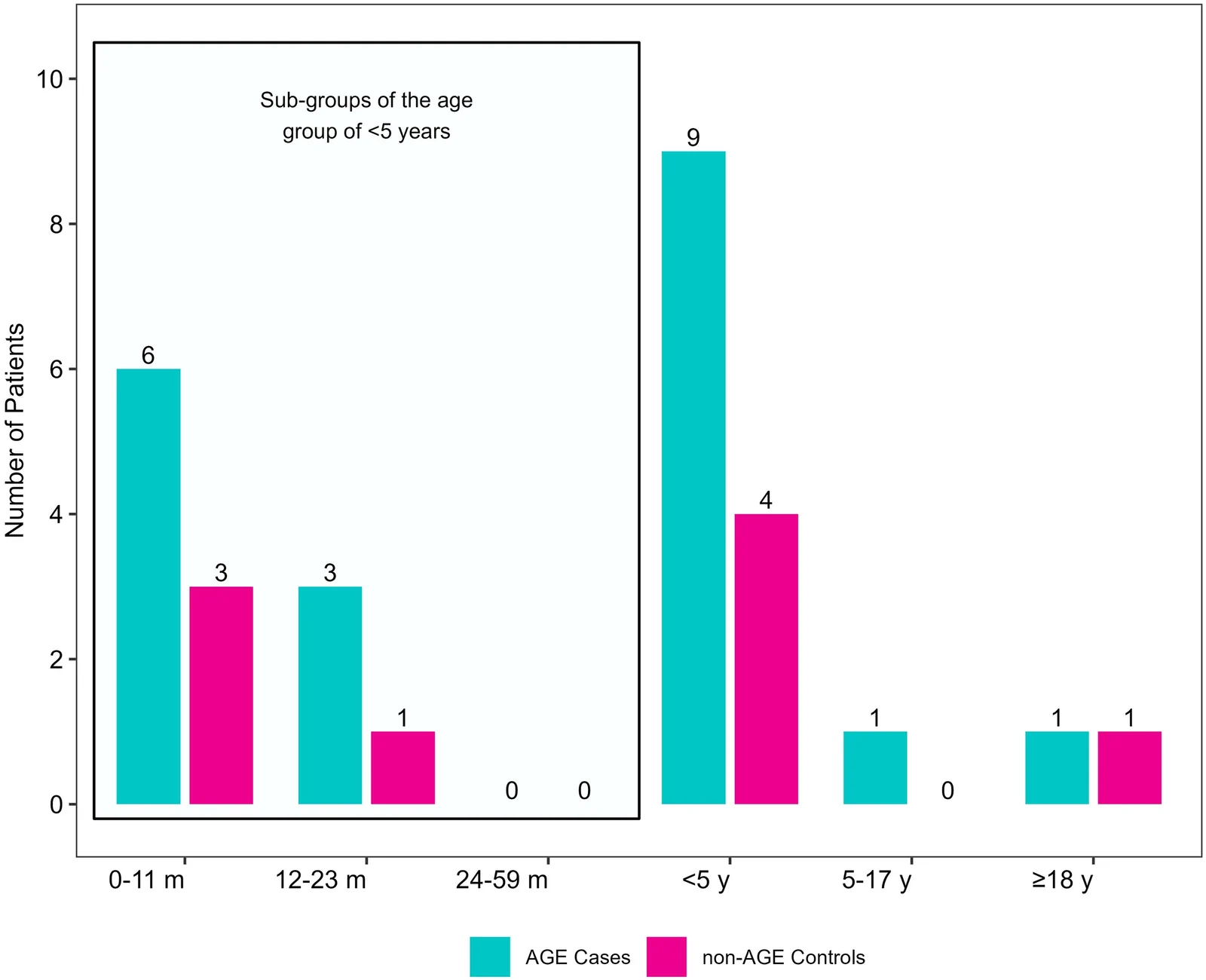
Age distribution of hospitalized norovirus-positive acute gastroenteritis (AGE) cases and non-AGE controls. Abbreviations: y, year; m, month.

**Table 1.** Demographic and Clinical Characteristics of Hospitalized Norovirus-Positive AGE Cases and Non-AGE Controls, January–September 2023

ID	Sex	Age (Months)	Duration of Illness (Days)	Greatest Number of Episodes of Diarrhea/24 h	Greatest Number of Episodes of Vomiting/24 h	Fever	Rehydration	Duration of Norovirus Shedding (Days)	Days Between Hospital Admission and First Specimen Collection
AGE Cases (n = 11)									
AC1	M	11	5	16	None	No	IV	29	1
AC2	M	6	6	20	None	No	IV	29	1
AC3	F	492 (41 y)	1	14	None	No	IV and oral	9.5	0
AC4	F	23	3	13	None	Yes	IV	49	2
AC5	M	4	4	14	None	No	IV	29	1
AC6	M	2	5	8	None	No	IV	9.5	1
AC7	M	9	5	10	None	Yes	IV	9.5	1
AC8	F	15	3	20	3	No	IV and oral	9.5	1
AC9	M	11	6	10	None	Yes	Oral	9.5	0
AC10	M	96 (8 y)	6	20	None	No	IV and oral	20	1
AC11	F	132 (11 y)	5	10	None	No	IV and oral	10	0
Summary % or Median (IQR)	Male: 64% Female: 36%	Median: 11 (6–96)	Median: 5 (3–6)	Median: 14 (10–20)	N/A	Yes: 27% No: 73%	Oral: 9% IV: 55% IV and Oral: 36%	Median: 10 (9.5–29)	Median: 1 (0–1)
Non-AGE Controls (n = 5)									
NAC1	F	516 (43 y)	N/A	N/A	N/A	No	N/A	9.5	1
NAC2	F	23	N/A	N/A	N/A	No	N/A	9.5	1
NAC3	M	4	N/A	N/A	N/A	No	N/A	49	2
NAC4	M	6	N/A	N/A	N/A	No	N/A	91.5	7
NAC5	M	12 (1 y)	N/A	N/A	N/A	No	N/A	9.5	6
Summary % or Median (IQR)	Male: 60% Female: 40%	Median: 12 (6–23)	N/A	N/A	N/A	No: 100%	N/A	Median: 9.5 (9.5–49)	Median: 2 (1–6)

Abbreviations: AGE, acute gastroenteritis; AC, AGE case; NAC, non-AGE control; M, male; F, female; IV, intravenous; IQR, interquartile range; N/A, not applicable.

Among AGE cases, the median of the maximum number of stools in a 24-hour period was 14 (IQR, 10–20), the median duration of illness was 5 days (IQR, 1–6 days), 3 (27%) had fever, and 10 (91%) needed intravenous rehydration. Stool specimens were collected a median of 1 day (IQR, 0–1 days) after admission for AGE cases and 2 days (IQR, 1–6 days) after admission for non-AGE controls.

Seven AGE cases and 3 non-AGE controls were enrolled in Kishoreganj, while 4 AGE cases and 2 non-AGE controls were enrolled in Rajshahi (Figure 2). Seventy-four of a maximum of 80 stool specimens (5 specimens from each of 16 study persons) were collected, representing 93% of the intended longitudinal samples. Twenty-eight of 74 (38%) specimens tested positive for norovirus. Of these, GII.4 Sydney[P16] was detected in 20 (71%) of 28 norovirus-positive specimens, 7 (25%) typed as GII untypeable, and 1 (4%) typed as GII.3[P16].

**Figure 2.**
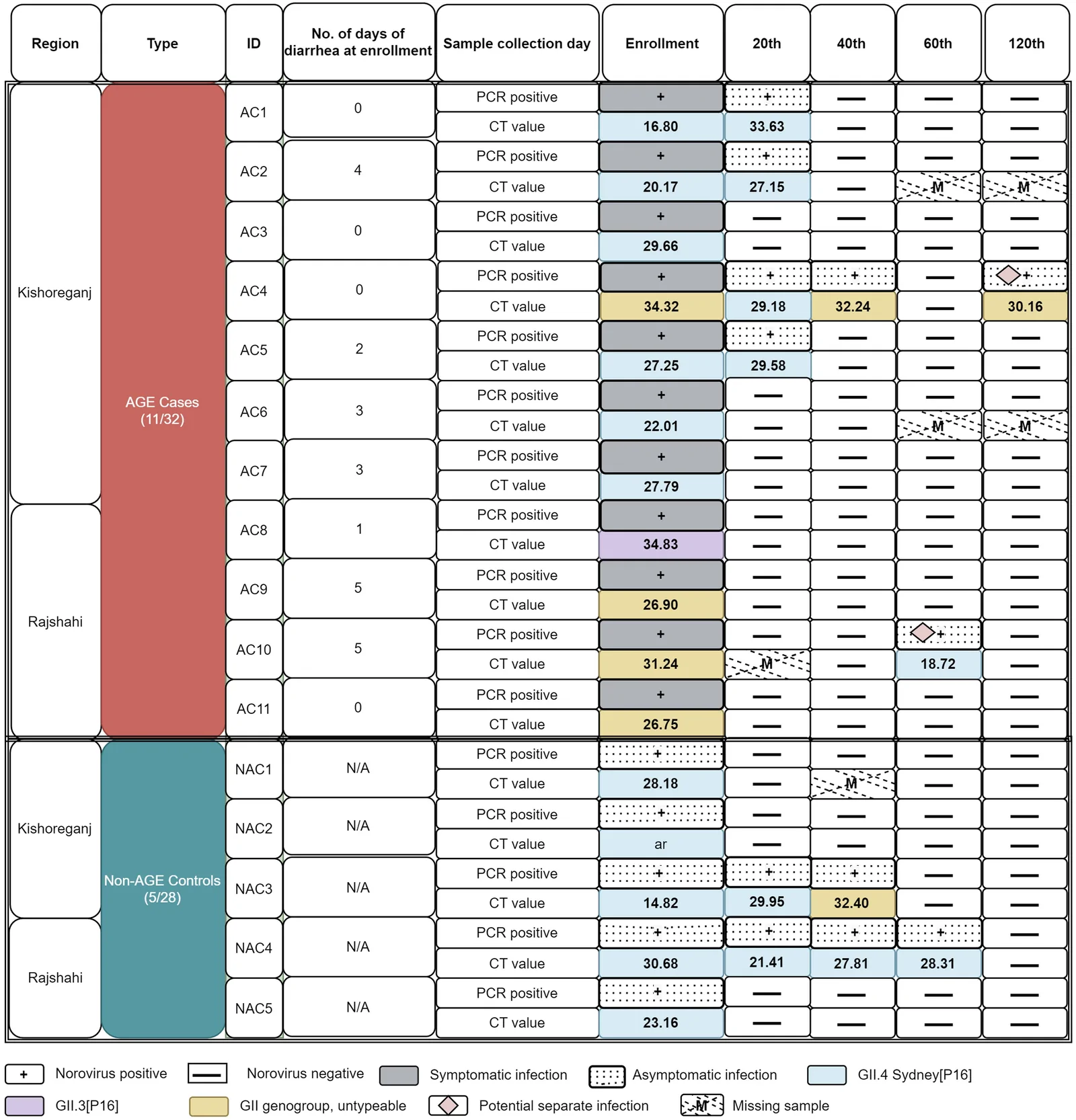
Course of the illness, duration of follow-up, number of specimens collected, and testing and genotyping results for each hospitalized acute gastroenteritis (AGE) case and non-AGE control. Note: The fecal specimen labeled as being collected at day 120 since enrollment from AC10 was obtained on day 142 since enrollment.

Within each of the 11 AGE cases (AC) and 5 non-AGE controls (NAC), all norovirus-positive specimens belonged to the same genogroup or genotype (Figure 2). Where available, we compared sequences from people with 2 or more norovirus-positive specimens (Table 2). Using a threshold of >99% sequence identity to define the same strain, most comparisons indicated that subsequent detections represented prolonged shedding rather than new infections. Two AGE cases (AC4 and AC10) presented with a negative specimen between 2 positive results, suggesting potential reinfections; however, only the first episode was included in the shedding duration analyses. None of the AGE cases and non-AGE controls reported AGE symptoms during subsequent shedding periods or upon possible reinfection.

**Table 2.** Pairwise Sequence Alignment of Norovirus-Positive Specimens From Person Hospitalized AGE Cases and Non-AGE Controls in Bangladesh

^a^Patient ID	Collection Day and Genotype of Specimen Pairs Being Compared				Pairwise Sequence Similarity (%)
AC1	Enrollment	GII.4 Sydney[P16]	20th	GII.4 Sydney[P16]	91.9
AC2	Enrollment	GII.4 Sydney[P16]	20th	GII.4 Sydney[P16]	91.9
AC5	Enrollment	GII.4 Sydney[P16]	20th	GII.4 Sydney[P16]	99.2
NAC3	Enrollment	GII.4 Sydney[P16]	20th	GII.4 Sydney[P16]	99.8
NAC4	Enrollment	GII.4 Sydney[P16]	20th	GII.4 Sydney[P16]	99.2
			40th	GII.4 Sydney[P16]	99.1
			60th	GII.4 Sydney[P16]	99.1
	20th	GII.4 Sydney[P16]	40th	GII.4 Sydney[P16]	99.8
			60th	GII.4 Sydney[P16]	99.8
	40th	GII.4 Sydney[P16]	60th	GII.4 Sydney[P16]	100

Abbreviations: AC, AGE case; NAC, non-AGE control.

^a^Three sequences for AC4 and 1 sequence each for AC10 and NAC3 could not be obtained, so these specific comparisons are not included.

The median duration of shedding was 10 days (IQR, 9.5–29 days) among AGE cases and 9.5 days (IQR 9.5–49 days) among non-AGE controls (log-rank test of equality *P* > .05) (Figure 3). Using 2 alternative definitions for the duration of shedding (narrow or wide definitions) revealed similar trends in the duration of shedding among AGE and non-AGE controls (Supplementary Figure 1).

**Figure 3.**
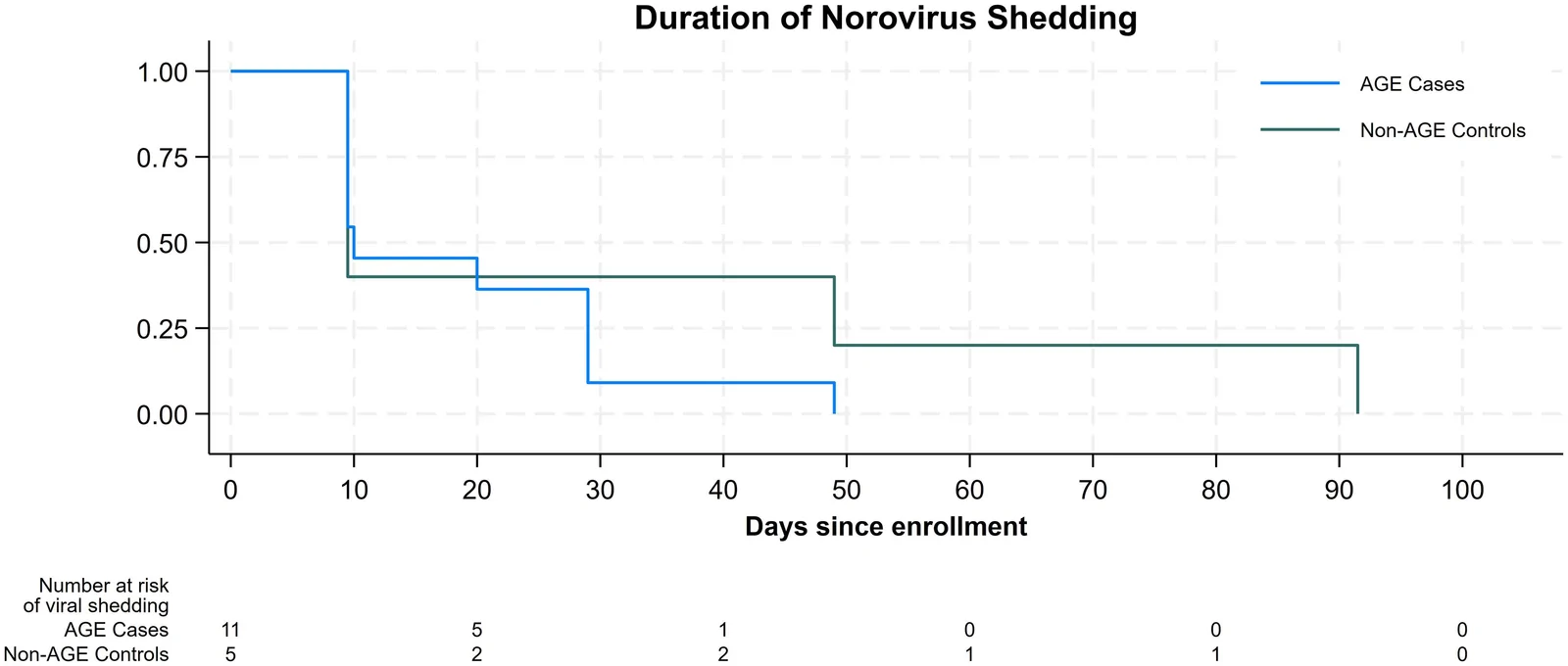
Kaplan–Meier estimates of duration of norovirus shedding since enrollment among acute gastroenteritis (AGE) cases (n = 11) and non-AGE controls (n = 5) in Bangladesh. There were no differences in median duration of shedding between cases and controls (long rank test of equality *P* > .05). Median shedding duration among cases was 10 d (interquartile range [IQR] 9.5–29) and among controls 9.5 d (IQR 9.5–49). The duration of shedding was calculated as the number of days since hospitalization until the midpoint between the last norovirus-positive test result and the first norovirus-negative test result.

Cycle threshold value distributions among AGE cases and non-AGE controls overlapped within specific sampling timeframes (Figure 4).

**Figure 4.**
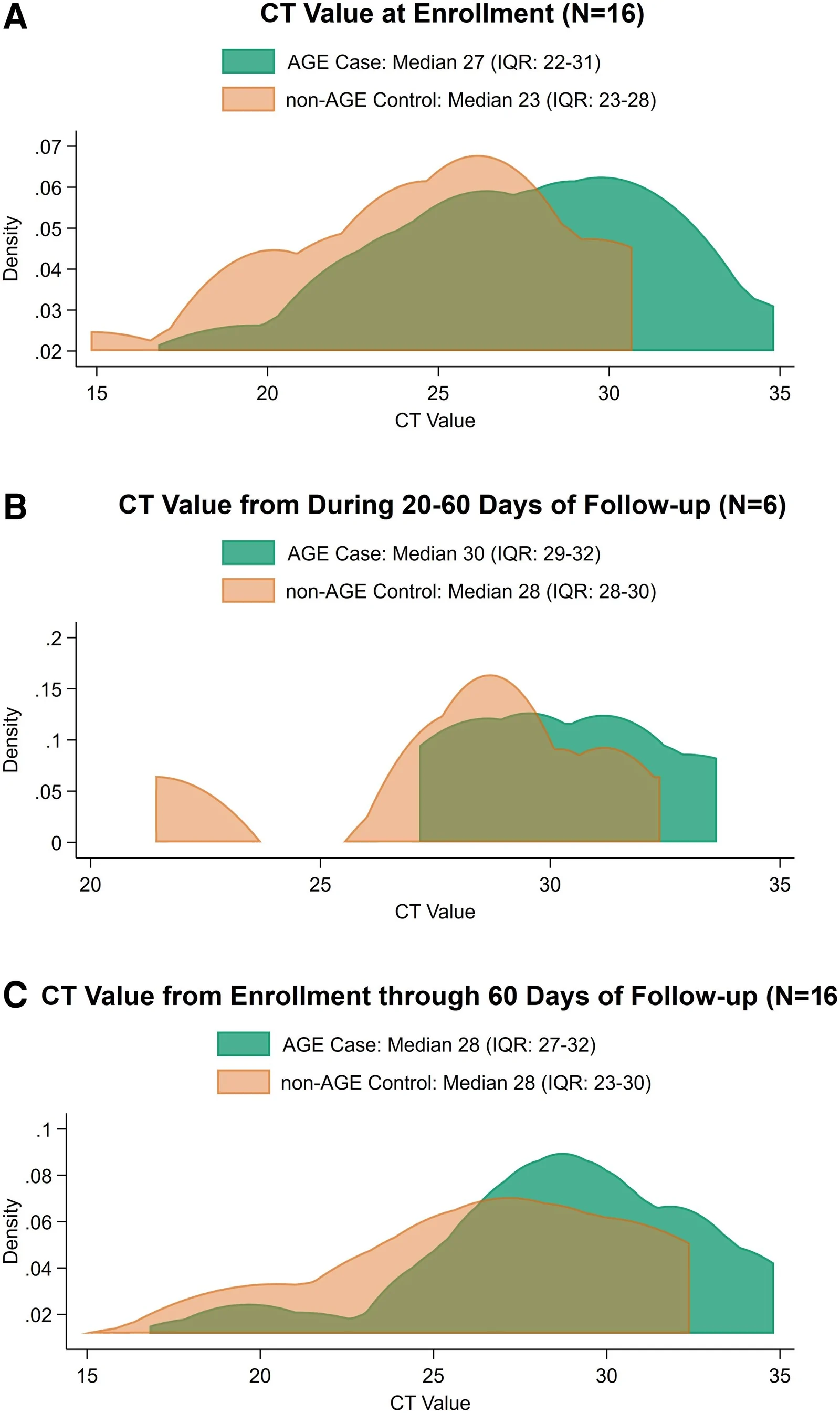
Distribution of GII norovirus cycle threshold (Ct) values among hospitalized norovirus-positive acute gastroenteritis (AGE) cases (N = 11) and non-AGE controls (N = 5). *A*, At enrollment, the distribution of Ct values is based on the enrollment samples from all 16 persons. *B*, During 20–60 d of follow-up, the distribution of Ct values is based on 5 samples from 4 AGE cases and 5 samples from 2 non-AGE controls. *C*, From enrollment through follow-up, the distribution of Ct values is based on 26 samples from all 16 persons.

## DISCUSSION

Little is known about the duration and magnitude of norovirus shedding among symptomatic and asymptomatic persons in highly endemic settings. In our study, shedding duration was broadly similar among AGE cases and non-AGE controls, matching observations from human challenge studies in high-income settings [16, 24, 25]. However, unlike human challenge studies that prospectively follow people after experimental infection, our controls had no AGE symptoms during the 14 days before enrollment and therefore were either truly asymptomatic or had recovered from AGE more than 14 days earlier. In either scenario, we expected shedding in non-AGE controls to be shorter than in AGE cases, who reported diarrhea symptoms at presentation and had illness onsets within 5 days of enrollment. Longer shedding periods have been reported in symptomatic compared with asymptomatic persons [3, 14], although the sample sizes for these comparisons, as in our study, were relatively small. Larger studies with detailed longitudinal monitoring of norovirus shedding following natural infection are needed to better characterize shedding kinetics in symptomatic and asymptomatic persons in highly endemic settings. Nonetheless, our findings show that asymptomatic or postsymptomatic norovirus shedding can be protracted (∼40–60 days). This supports the notion that prolonged shedding might contribute, at least in part, to the high prevalence of norovirus detected among asymptomatic persons in Bangladesh.

The amount of norovirus detected over the course of infection was comparable between AGE cases and non-AGE controls, consistent with earlier studies reporting similar cumulative norovirus excretion in symptomatic and asymptomatic persons [13, 19, 24, 25]. At the person level, the majority of AGE cases and non-AGE controls had an expected gradual decline in norovirus viral load after symptom resolution or during asymptomatic infections. However, 2 persons (AC4 and NAC4) exhibited fluctuations in viral load over the 0–40-day follow-up period. Although these fluctuations could be attributed to new infections, intraperson variability in the quantity of noroviruses shed in stool over the course of single infections has been shown previously [3, 13, 16, 24], and NAC4 had the same genotype (and P-type) and high sequence identity (>99%) in all specimens. We also observed high degree of variability in the quantity of norovirus shed in stool from person to person, as has been noted previously [13]. This variability in the viral load within and between persons could be related to differences in stool specimen consistency or volume and logistical factors, such as the timing of specimen collection, handling, and storage conditions [26, 27]. Our findings highlight the difficulty in determining an optimum Ct value cutoff to identify clinically relevant norovirus infections [28] and suggest potential limitations in using Ct values alone to estimate the contribution of norovirus to diarrhea in certain settings.

Understanding norovirus shedding duration can help guide selection criteria for (symptomatic) cases and (asymptomatic) controls in case–control studies assessing the burden of norovirus diarrhea. Our study protocol included non-AGE controls based on a 14-day symptom-free window, but 25% of our AGE cases were positive for norovirus for ∼30 or more days after enrollment. Consequently, we might have inadvertently enrolled persons still shedding norovirus from a recent symptomatic episode in our non-AGE control group. Other studies have noted the median duration of norovirus shedding to be 3–4 weeks [13, 16]; thus, a longer symptom-free period could be considered for controls to better distinguish between active and resolved infections. Further, applying similar asymptomatic criteria during the enrollment of symptomatic cases might also improve case–control comparisons, as previously suggested [29].

The detection of norovirus at similar concentrations in asymptomatic compared to symptomatic persons, as well as the sustained shedding observed after clinical recovery and during asymptomatic infections, suggests that asymptomatic persons may act as reservoirs for norovirus, unknowingly shedding the virus and contributing to community transmission, particularly given the low infectious dose required to cause disease [30]. However, detection of norovirus RNA does not necessarily indicate the presence of infectious virus, and additional studies could focus on confirming if norovirus-positive stools in otherwise healthy controls are infectious using the human intestinal enteroid platform [31]. Similarly, additional epidemiological studies, eg, household transmission studies, are needed to establish the relationship between prolonged viral shedding and onward transmission.

Our study has several limitations. Although our results align with previous studies [13, 16, 19, 24, 25], the findings are limited by the small sample size and should be interpreted accordingly. Our study design and definitions could have biased the duration of shedding in several ways. As initial stool samples were collected at enrollment, the first norovirus-positive result does not reflect the actual start of viral shedding, possibly underestimating the total duration of shedding. Similarly, as specimens were collected at fairly wide intervals, the last positive or first negative norovirus test result is not indicative of the end of viral shedding and can underestimate or overestimate total duration. For this reason, we defined the end of shedding as the midpoint between these sampling dates. Alternate definitions capturing a narrow and a wide shedding period showed similar trends in the duration of shedding (Supplementary Figure 1), but frequent weekly sampling would have improved precision. We considered the possibility of new infections during follow-up, but several specimens could not be genotyped, so in 2 instances (AC4 and NAC3), we were unable to distinguish between new infections and continued shedding. Only 7% of stool specimens were not collected, but for 1 AGE case (AC10), a missing result before their first norovirus-negative specimen introduced uncertainty about the duration of shedding. If that missing sample was negative, we might have overestimated shedding duration in this person. For 2 AGE cases (AC1 and AC2), pairwise sequence alignment showed 91.9% identity between specimens, suggesting divergent variants and potentially 2 separate infections, so we might have overestimated shedding duration in these 2 AGE cases. Rapid antigen detection test results correlated poorly with confirmatory RT-qPCR testing, limiting our ability to efficiently identify norovirus-positive AGE cases and non-AGE cases for additional follow-up. Finally, although absolute quantification using a validated standard curve would provide a more precise measure of norovirus RNA concentration, our primary objective was to characterize shedding duration among symptomatic and asymptomatic individuals. Consequently, we utilized Ct values as an inverse proxy of viral load, rather than estimating absolute RNA copy numbers.

In summary, our study aimed to capture the dynamics of norovirus excretion in stool over time and demonstrates that the quantity and duration of norovirus shedding can be similar between people with and without AGE symptoms in Bangladesh. Our findings have several potential implications. First, long-term viral shedding may influence the higher prevalence of norovirus detected among non-AGE controls compared with AGE cases [9]. Second, there may be limitations in using Ct values to attribute diarrhea to norovirus at both the individual and population levels in high-burden settings. Third, exclusion criteria for cases and controls might need to be refined in case–control studies assessing the burden of norovirus. Finally, asymptomatic shedding may play a role as a source of norovirus infection and transmission. Future studies should incorporate larger sample sizes with more frequent testing to better estimate shedding duration and infectiousness, which will inform public health interventions, particularly in high-transmission, resource-limited settings.

## Supplementary Material

ofag658_Supplementary_Data

## Supplementary Data

Supplementary materials are available at *Open Forum Infectious Diseases* online. Consisting of data provided by the authors to benefit the reader, the posted materials are not copyedited and are the sole responsibility of the authors, so questions or comments should be addressed to the corresponding author.
